# Headache in School Children

**Published:** 1881-02

**Authors:** 


### HEADACHE IN SCHOOL CHILDREN.

A recent writer, Dr. Triechler, states that about one-third of the pupils in schools suffer more or less from headache. It leads to poorness of blood, and loss of cheerfulness and mental energy. Its chief cause is probably overwork, and especially nocturnal study. The anatomical changes which accompany the more advanced stages of this habitual headache are, in the author's opinion: 1. Trophic changes in the ganglion-cells of the brain cortex, caused by anæmia. An anæmic brain is much more easily exhausted by mental exertion than a normal one. 2. Passive dilatation of the cerebral blood vessels and consequent stasis ; the perivascular spaces round the capillaries become narrowed, the removal of waste products is thus hindered, and in this way again trophic disturbance is caused. Recent views, which regard progressive paralysis as commencing by vaso motor trophic changes in the brain cortex, paretic dilation of the vessels of the pia mater, and degeneration of the cortex through lymph-stasis, increase the significance and importance of the conditions believed by the author to be brought about by prolonged habitual headache in young people.

